# Provision of breast cancer care and survival in Germany – results from a population-based high resolution study from Saarland

**DOI:** 10.1186/1471-2407-14-757

**Published:** 2014-10-10

**Authors:** Bernd Holleczek, Hermann Brenner

**Affiliations:** Division of Clinical Epidemiology and Aging Research, German Cancer Research Center, Im Neuenheimer Feld 581, 69120 Heidelberg, Germany; Saarland Cancer Registry, Präsident Baltz-Straße 5, 66119 Saarbrücken, Germany; German Cancer Consortium (DKTK), Im Neuenheimer Feld 280, 69120 Heidelberg, Germany

**Keywords:** Breast cancer, Clinical Practice Guidelines, Evaluation of cancer care, Population-based cancer registry, Relative survival, Germany

## Abstract

**Background:**

Studies on the implementation of Clinical Practice Guidelines (CPG) and particularly its effect on breast cancer (BRC) survival on a population-level are scant. This population-based high resolution study from Germany aims at providing data on the usage of BRC treatment, the extent of adherence to CPG and, as a novelty, survival of BRC patients according to major recommended treatment options.

**Methods:**

Data from the Saarland Cancer Registry including women diagnosed with invasive BRC without distant metastasis and followed up between 2000 and 2009 were used. Provision of cancer care according to major treatment options is presented by age, clinical subtypes of BRC, and over time. Conventional and modeled period analysis was used to derive estimates of most up-to-date 5-year relative survival (RS) and the effect of non-adherence to CPG on relative excess risk of death (RER).

**Results:**

The study revealed increasing guideline adherence, with high levels already seen for local treatment (e.g. 67% of the BRC patients in 2008/09 received breast conserving surgery), and substantial progress since the millennium change with regard to sentinel node dissection (SND) and adjuvant systemic treatments (e.g. SND and chemotherapy provided to 62% of all patients and 79% of the patients with nodal positive or hormone receptor negative BRC in 2008/09, respectively). It further demonstrated increased cancer related mortality among patients without guideline compliant cancer treatment (e.g. patients with nodal positive and hormone receptor negative BRC who were not treated with chemotherapy had a 5-year RS of 29% (RER: 2.89, 95% CI: 1.46–5.71) compared to 54% for patients obtaining chemotherapy).

**Conclusions:**

This study provides data on the implementation of CPG in a highly developed European country and extends available population-based survival data of BRC patients and may provide evidence of increased cancer related excess mortality, if BRC patients do not receive guideline compatible treatment.

## Background

Invasive breast cancer (BRC) is the most frequent cancer among women worldwide [1], with estimated 72,000 new cases and 17,200 deaths in Germany in 2008 [2]. Within the past two decades, BRC mortality has steadily decreased as a result of therapeutic improvements and increased early detection [3, 4]. However, there is ongoing research and controversy with regard to the possible size of the effect of early detection and organized mammography screening programs in developed countries [5–9].

Population-based survival studies of BRC patients with regard to delivered treatment according to available Clinical Practice Guidelines (GPG) are scant. In the past, population-based data on long-term survival of BRC patients have commonly been restricted to overall estimates or provided estimates with regard to age, tumor stage and further major prognostic factors only [10–15]. Clinicians, patients, researchers and health care planners are in great need for unselected survival data, which may present – along with data on the actual usage of cancer specific treatments – an “overall” picture of cancer related excess mortality according to cancer treatment options, and provide important information on the effectiveness of cancer care in the community setting.

Survival studies using available routine data from population-based cancer registries are intrinsically difficult to interpret with regard to effects of early detection and postponement of death of the patients due to effective treatment, as these registries commonly collect only very basic information items only (e.g. sex of the patient, cancer site, date of diagnosis, summary stage, tumor morphology, and follow-up of the patient) [16, 17]. As a prerequisite for explaining observed survival differences with regard to effects from early detection and cancer treatment, population-based cancer registries need to additionally collect detailed information on the stage at diagnosis, the diagnostic workup and administered treatment of the patients. Survival studies which are based on such extended data are often termed as ‘high resolution’ studies [16].

This high resolution study from Germany aims at providing population-based data on the extent and variation of the delivery of BRC treatment and adherence to available CPG in a highly developed European country. It further extends a previously published study with detailed cancer survival by cancer characteristics for clinical subgroups of patients [15] and provides survival of BRC patients with regard to the effect of non-adherence to CPG on cancer related mortality on a population-level within the calendar period 2000–2009.

## Methods

This study used data from the Saarland Cancer Registry, which covers the federal state of Saarland in South-Western Germany with a population of approximately 1.02 million inhabitants in 2009 (constituting 1.3% of the national population). The registry collects information on invasive and in situ neoplasms since 1968 and obtains notifications from hospitals, radiotherapy departments, pathology laboratories, screening programs and general practitioners. The proportion of the registered incident cancer cases is regularly estimated to be >95% [2, 18].

The study included 8571 female patients with an invasive BRC (ICD-10: C50) diagnosed between 2000 and 2009 and aged ≥15 years. Patients with a previous invasive BRC were not included. Mortality follow-up was based on death certificates and linkage of patients with central population registries (see [15] for details). In addition to routinely collected tumor information, data on further tumor characteristics (such as HR status, or HER2/neu expression) and cancer treatment were obtained by means of requested additional reports and data collection at source by registry staff based on a standardized extraction protocol (see [15] for details). Standard procedures of quality control of the registry were applied with respect to accuracy, completeness and consistency of the extended data [19]. For patients diagnosed with a bilateral BRC, tumor and treatment data of the more advanced tumor were included into the study database.

For the analyses, three age categories were used: 15–49, 50–69 and ≥70 years. The European Network of Cancer Registries recommendations were used to classify tumor stage as “localized” (T1-3N0M0), “regionally or locally advanced” (T1-3 N + M0, T4M0), “distant metastasis” (M1), or “missing” [20]. Histologic grade included the categories “low”, “intermediate”, “high”, and “missing” according to the WHO scheme. HR status was classified as “positive” (both estrogen and progesterone receptor positive), “mixed” (either estrogen or progesterone receptor positive), “negative” (both estrogen and progesterone receptor negative), and “missing”. The categories of HER2/neu expression were “positive” (including borderline), “negative”, and “missing”.

The currently available German national CPG on the treatment of BRC [21], which differ only marginally from guidelines from other countries [22], recommend breast conserving surgery (BCS) followed by radiotherapy or mastectomy alone for effective local treatment of early stage BRC or mastectomy and radiotherapy for locally advanced tumors. Dissection of lymph nodes (preferably sentinel node dissection) is required for proper staging. Adjuvant systemic treatment aims at preventing tumor recurrence and includes chemotherapy according to stage and risk of recurrence, antiestrogen treatment for patients with hormone receptor (HR) positive or mixed tumors, and monoclonal antibodies (trastuzumab) if a carcinoma shows HER2/neu expression. In case of advanced disease, systemic treatment is recommended prior to surgery. Elderly patients should receive an adjuvant systemic treatment comparable to younger patients, taking into account altered organ functions and comorbidity.

For local surgery, the categories “BCS” including lumpectomy and quadrantectomy, “mastectomy”, “none”, and “missing” were used. Dissection of lymph nodes was either categorized as “axillary lymph node dissection” (ALND), “sentinel node dissection” (SND), “ALND after SND”, “none”, and “missing”. To categorize the delivery of adjuvant radiotherapy and systemic treatments, the categories “yes”, “none”, and “missing” were used.

The following major treatment options according to the national CPG [21] were evaluated with regard to the provision to and the survival of the BRC patients without distant metastases: local treatment consisting of (i) either BCS and radiotherapy or mastectomy alone in patients with T1/T2N0 tumors, (ii) BCS or mastectomy followed by radiotherapy in patients with T1/T2N + tumors, (iii) combination of mastectomy and radiotherapy in patients with T3/T4 tumors, (iv) dissection of lymph nodes (SND, ALND after SND and ALND), and systemic treatment consisting of (v) chemotherapy in patients with nodal positive or HR negative BRC, (vi) antiestrogen treatment in patients with HR positive or mixed tumors, and (vii) trastuzumab given to patients with HER2/neu expressed tumors.

Univariate description of the patients and tumor characteristics was derived for the calendar intervals 2000–2004 and 2005–2009. The provision of cancer care by age and temporal trends was analyzed for the calendar intervals 2005–2009 and 2000–2009, respectively.

Relative survival (RS), which quantifies excess mortality due to the cancer and captures both direct and indirect mortality is derived as ratio of observed survival of the cancer patients to expected survival of a group of sex-, age- and calendar time of observation-matched individuals with average risk of death from all causes from the source population [23]. The Ederer II method was used for deriving expected survival estimates [24]. Details on the generation of the used life tables may be found elsewhere [25].

Period analysis methods were used to obtain up-to-date estimates of 5-year RS. Classical cohort based survival estimates may reflect possible recent progress in cancer care to a very limited extent only and thus lag behind the expected survival of most recently diagnosed cancer patients [26, 27]. Period analysis uses survival experience observed in a specified calendar period (typically, the most recent period with available incidence and mortality follow-up information), and, in addition to right censoring, survival observations are left truncated at the beginning of the calendar period (e.g. a period estimate of 5-year survival derived for the calendar period 2005–2009 may include patients diagnosed between 2000 (at the earliest) and 2009, and therefore, the number of patients contributing survival experience to a period estimate differs from the number of subjects diagnosed in the respective calendar period) [28].

Period estimates of 5-year RS of patients were derived by age, tumor characteristics, and recommended local and systemic cancer treatment. Patients without follow-up information and with death certificate only (DCO) notified tumors were excluded from the survival analyses. Standard errors are based on Greenwood’s method [29]. Age standardized survival was derived as weighted average of age group-specific survival according to the International Cancer Survival Standards (ICSS) [30].

Model-based period analysis was used as previously described to quantify relative excess risks of death (RER) and for statistical significance testing [31, 32]. Based on an additive hazards model, RER quantifies the relative cancer related excess mortality between the specific “exposed” groups of cancer patients (defined by age, stage, other characteristics, or cancer treatment) compared to the “unexposed” reference group of matched persons from the general population [33, 34]. The models to investigate the effect of guideline adherence on 5-year RS and RER for different groups of patients with regard to tumor characteristics and treatment options included the information, whether a patient received the investigated treatment (e.g. antiestrogen treatment in patients with HR positive or mixed tumors) as dichotomous variable and adjusted for age, T, N, histologic grade, hormone receptor status and HER2/neu expression (explanatory variables of categorical type). For the RER estimates, 95% confidence intervals (CI) were derived. The reported p-values are based on Wald tests (based on an asymptotic Chi-squared statistic) for inclusion of the respective variables into the models.

The R Language and Environment for Statistical Computing (release 2.11.1) [35] and the “periodR” package (release 1.0-6) were used for the data preparation, survival estimation and modeling [36, 37].

The data used for this study were collected by the Saarland Cancer Registry according to state legislation for the purpose of monitoring cancer care and outcomes and the anonymized data were used according to the respective provisions for the use of research data.

## Results

Table 1 shows characteristics of the included 8571 patients and their tumors. In 2005–2009, the patients were on average 63.3 years old at diagnosis, 56% had a tumor which was 2 cm or larger, 44% presented with positive lymph nodes and 9% with distant metastases. Most frequently, tumors were of intermediate grade (68%), HR positive (72%) and HER2/neu negative (76%). Almost all tumors were microscopically confirmed. Follow-up was available for virtually all patients and 2% of the tumors were notified by a death certificate only. Overall, very similar characteristics were seen for patients diagnosed in 2000–2004 and 2005–2009.

**Table 1:** **Characteristics of female breast cancer patients** (**ICD**-**10**: **C50**) **from Saarland diagnosed between 2000 and 2009**

Characteristic	Category	2000-2004		2005-2009	
		n	%	n	%
Overall		4147		4424	
Age	15-49 years	754	18.2	785	17.7
	50-69 years	1973	47.6	2098	47.4
	> = 70 years	1420	34.2	1541	34.8
T	Available	3880	93.6	3951	89.3
	1^a^	1671	43.1	1742	44.1
	2^a^	1574	40.6	1633	41.3
	3^a^	206	5.3	247	6.3
	4^a^	429	11.1	329	8.3
N	Available	3483	84.0	3650	82.5
	0^a^	1964	56.4	2057	56.4
	1^a^	1159	33.3	1017	27.9
	2^a^	273	7.8	345	9.5
	3^a^	87	2.5	231	6.3
M	Available	3458	83.4	3222	72.8
	0^a^	3129	90.5	2923	90.7
	1^a^	329	9.5	299	9.3
Microscopic confirmation		4031	97.2	4312	97.5
Histologic grade	Available	3856	93.0	4201	95.0
	Low^a^	277	7.2	304	7.2
	Intermediate^a^	2273	58.9	2872	68.4
	High^a^	1306	33.9	1025	24.4
Hormone receptor status	Available	3620	87.3	3720	84.1
	Positive (ER + PgR+)^a^	2474	68.3	2688	72.3
	Mixed (ER + or PgR+)^a^	531	14.7	451	12.1
	Negative (ER- PgR-)^a^	615	17.0	581	15.6
HER2/neu expression	Available	2505	60.4	3598	81.3
	Positive^a,b^	625	25.0	866	24.1
	Negative^a^	1880	75.0	2732	75.9
Death certificate only notified		67	1.6	100	2.3
No follow-up available		79	1.9	48	1.1

*ER*: estrogen receptor; *PgR*: progesterone receptor; a) proportions among patients with available information; b) including 401 tumors with borderline expression; the table is based on previously published data [15].

Provision of local treatment, lymph node dissection and adjuvant systemic treatment to BRC patients presenting without distant metastases and who were diagnosed between 2005 and 2009 is shown in Table 2. Information on local treatment and lymph node dissection was available for about 76% of the patients. BCS with or without radiotherapy was given to 57% and 10% of the patients. Overall, 14% and 18% of the patients underwent a mastectomy with or without radiotherapy, respectively. Staging was based on SND, ALND after SND and ALND for 37%, 17% and 42% of the patients respectively. Almost all patients received local surgery and dissection of lymph nodes (the proportions of patients without such treatment were below 2% each).

**Table 2:** **Provision of cancer care to breast cancer patients without distant metastasis by age**

Treatment	Category	Overall		15-69 years		> = 70 years	
		N	%	N	%	N	%
Overall		4025		2691		1334	
Local treatment	Available	3038	75.5	2078	77.2	960	72.0
	BCS^a^	316	10.4	187	9.0	129	13.4
	BCS + radiotherapy^a^	1716	56.5	1342	64.6	374	39.0
	Mastectomy^a^	558	18.4	262	12.6	296	30.8
	Mastectomy + radiotherapy^a^	424	14.0	284	13.7	140	14.6
	No surgery^a^	24	0.8	3	0.1	21	2.2
Lymph node dissection	Available	3043	75.6	2145	79.7	898	67.3
	ALND^a^	1274	41.9	812	37.9	462	51.4
	SND^a^	1139	37.4	849	39.6	290	32.3
	SND + ALND^a^	525	17.3	432	20.1	93	10.4
	None^a^	47	1.5	7	0.3	40	4.5
Chemotherapy^b^	Available	1571	86.2	1154	91.4	417	74.5
	Yes^a^	1215	77.3	1050	91.0	165	39.6
	None^a^	356	22.7	104	9.0	252	60.4
Antiestrogen treatment^c^	Available	2232	75.8	1556	80.0	676	67.6
	Yes^a^	2035	91.2	1433	92.1	602	89.1
	None^a^	197	8.8	123	7.9	73	10.9
Targeted therapy^d^	Available	443	55.7	325	58.9	118	48.4
	Yes^a^	188	42.4	164	50.5	24	20.3
	None^a^	255	57.6	161	49.5	94	79.7

Local treatment, lymph node dissection and adjuvant systemic treatment of female BRC patients without distant metastases (ICD-10: C50) from Saarland diagnosed between 2005 and 2009. *BCS*: breast conserving surgery; *ALND*: axillary lymph node dissection; *SND*: sentinel node dissection; a) proportions among patients with available information; b) among patients with positive lymph nodes or hormone receptor negative tumors; c) among patients with hormone receptor positive or mixed tumors; d) among patients with HER2/neu expressed tumors; patients presenting with distant metastases or DCO notified tumors were excluded.

**Figure 1:**
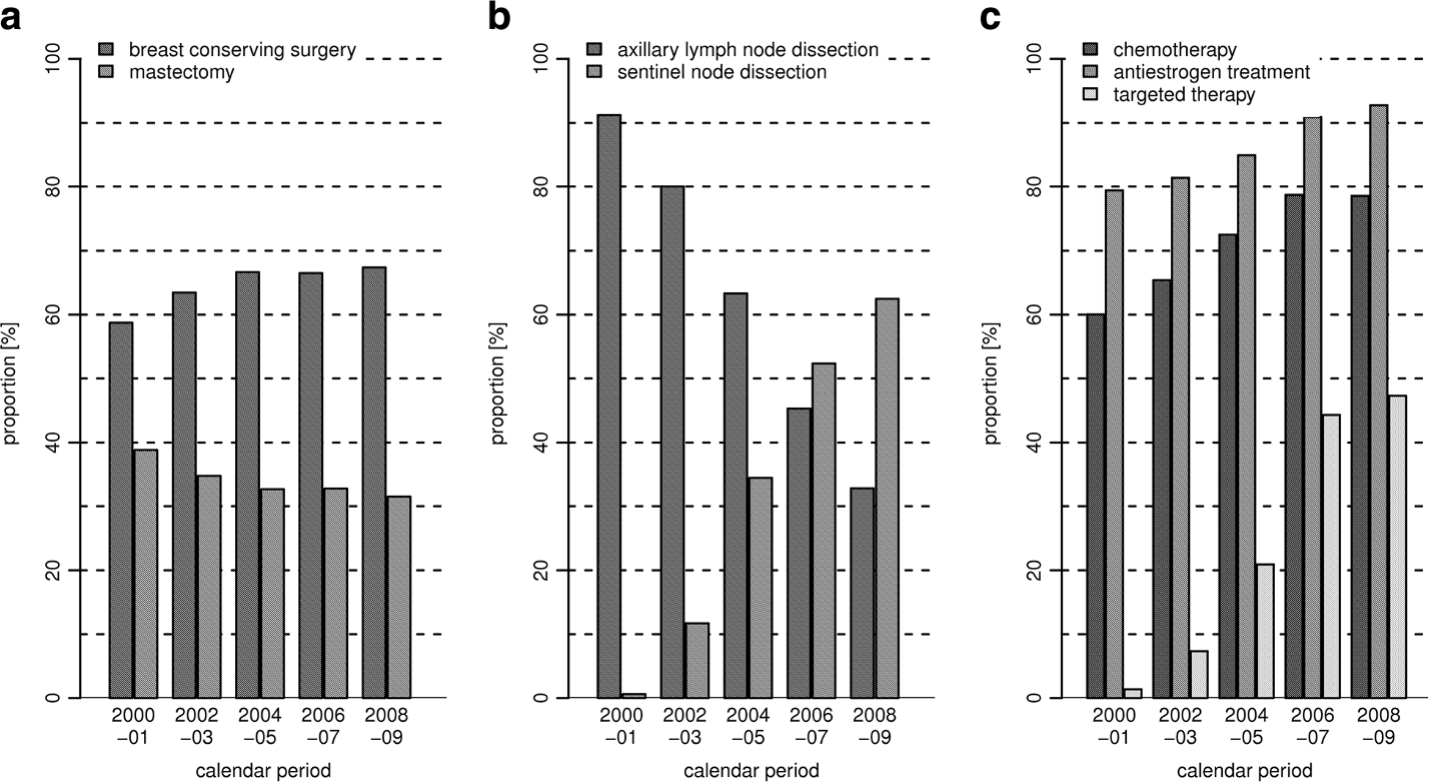
**Trends of the provision of cancer treatment between 2000 and 2009.** Trends of the provision of local treatment **(a)**, dissection of lymph nodes **(b)** and adjuvant systemic treatment (chemotherapy among patients with nodal positive or hormone receptor negative tumors; antiestrogen treatment among patients with hormone receptor positive or mixed tumors; trastuzumab among patients with HER2/neu expressed tumors; **c)** among female breast cancer patients without distant metastases (ICD-10: C50) from Saarland diagnosed between 2000 and 2009.

Of BRC patients with positive lymph nodes or HR negative tumors, HR positive or mixed tumors or HER2/neu expressed BRC, 77% received chemotherapy, 91% an antiestrogen treatment and 42% a targeted therapy, respectively. Comparison by age showed, that BRC patients aged ≥70 years received BCS with radiotherapy less often and mastectomy more often than younger patients (39% vs. 65% and 45% vs. 26%, respectively). SND was performed less often among elderly patients, too (43% vs. 60%). Likewise, the usage of chemotherapy and trastuzumab was higher among younger patients (91% vs. 40% and 51% vs. 20%, respectively). By contrast, antiestrogen treatment was quite similar in both age groups (92% and 89%, respectively).Figure 1 depicts time trends of the provision of major treatment options to BRC patients without distant metastases diagnosed between 2000 and 2009. During the study period, the usage of BCS increased from 59% in 2000/01 to 67% in 2008/09, whereas the proportion of patients with mastectomy dropped for about the same amount. Simultaneous to the tremendous rise of the usage of SND to 62%, the usage of ALND dropped from 91% to 33%. Chemotherapy usage among nodal positive or HR negative BRC patients increased from 60% to 79%, the usage of antiestrogen treatment among patients with HR positive or mixed tumors rose from 79% to 93%, and the proportion of patients with HER2/neu positive tumors who were given trastuzumab rose to 47% in 2008/09.

Table 3 presents 5-year RS in 2005–2009 by age and tumor characteristics. Overall age standardized 5-year RS was 83%. Survival of patients aged 70 years or older was substantially lower than survival observed for younger patients (survival was 89%, 88% and 77% for patients aged 15–49, 50–69, and ≥70 years, respectively). Five-year RS of patients with localized, locally/regionally advanced and metastasized BRC was 99%, 80%, and 23%. Survival further decreased with increasing grade (survival was 102%, 87% and 73%, for patients with low, intermediate and high grade tumors, respectively). Patients with HR positive or mixed tumors had a substantially higher survival (89% and 83%, respectively) compared to those patients with HR negative tumors (65%). If tumors showed HER2/neu expression, the patients’ survival was lower compared to patients with HER2/neu negative tumors (79% and 86%, respectively).

**Table 3:** **Five year relative survival of female breast cancer patients by age and tumor characteristics**

Characteristic	Category	N	RS ^a^	SE
Overall		7622	83.2	0.9
Age	15-49 years	1424	89.1	1.2
	50-69 years	3761	88.0	0.9
	> = 70 years	2437	76.7	1.8
Stage	Localized	3488	99.2	1.3
	Localized; T1	2110	104.9	1.6
	Localized; T2	1254	92.7	2.1
	Regionally/locally advanced	2688	79.8	1.6
	Regionally/locally advanced; T1/T2	2012	86.3	2.0
	Regionally/locally advanced; T3/T4	629	64.6	3.0
	Distant metastasis	414	23.1	2.8
	Missing	1032	81.7	2.1
Histologic grade^b^	Low	543	102.0	3.2
	Intermediate	4829	86.7	1.1
	High	2013	73.3	1.8
	Missing	221	65.0	5.0
Hormone receptor status^b^	Positive	4824	88.6	1.1
	Mixed	862	83.3	2.7
	Negative	1034	65.0	2.6
	Missing	886	77.7	2.7
HER2/neu expression^b^	Negative	4345	86.3	1.2
	Positive	1374	79.4	2.2
	Missing	1887	79.8	1.9

Five year RS of female breast cancer patients (ICD-10: C50) from Saarland estimated for the calendar period 2005–2009 by age and tumor characteristics. *N*: number of patients contributing survival experience; RS: point estimate of 5-year relative survival; *SE*: standard error of RS; a) except for age group-specific survival estimates, age standardized estimates of 5-year RS were derived using the ICSS weights; b) cases without microscopic verification were excluded.

Table 4 presents 5-year RS of BRC patients without distant metastases by age, tumor stage and local treatment. Five-year RS of patients receiving BCS without radiotherapy was 84%, whereas almost no decreased survival was observed for BRC patients receiving radiotherapy. Patients with mastectomy and mastectomy with radiotherapy had a 5-year RS of 88% and 78%, respectively. Patients without surgery had a 5-year RS of 41% only. For patients with localized T1/T2 tumors receiving a BCS followed by radiotherapy or mastectomy alone almost no decrease in 5-year RS was observed. Those with T1/T2 tumors with positive lymph nodes who received either BCS or mastectomy and additional radiotherapy had a 5-year RS of 90%. Five-year RS of patients with T3/T4 tumors who received mastectomy and radiotherapy was 89% if having clear lymph nodes compared to 74%, if having positive lymph nodes.

**Table 4:** **Five year relative survival of female breast cancer patients by age**, **tumor stage and local treatment**

Patient/tumor characteristics	Treatment	N	RS ^a^	SE
All patients	BCS	119	84.3	4.7
	BCS + radiotherapy	3285	98.5	1.5
	Mastectomy	613	87.7	2.9
	mastectomy + radiotherapy	736	78.4	3.3
	no surgery	43	40.8	6.7
	Missing	2406	84.1	1.4
T1/T2 tumors, nodal negative	BCS + radiotherapy or mastectomy	2467	99.5	1.6
T1/T2 tumors, nodal negative, 15-49 years		488	96.4	1.4
T1/T2 tumors, nodal negative, 50-69 years		1391	99.7	0.9
T1/T2 tumors, nodal negative, 70+ years		588	96.7	3.1
T1/T2 tumors, nodal positive	BCS + radiotherapy or mastectomy + radiotherapy	1272	90.1	2.7
T1/T2 tumors, nodal positive, 15-49 years		316	84.8	3.1
T1/T2 tumors, nodal positive, 50-69 years		697	89.5	2.0
T1/T2 tumors, nodal positive, 70+ years		259	94.8	4.9
T3/T4; nodal negative	mastectomy + radiotherapy	55	88.7	12.9
T3/T4; nodal positive	mastectomy + radiotherapy	256	73.6	5.1

Five year RS of female BRC patients (ICD-10: C50) from Saarland estimated for the calendar period 2005–2009 by age, tumor stage and local treatment. *N*: number of patients contributing survival experience; RS: point estimate of 5-year relative survival; *SE*: standard error of RS; a) except for age group-specific survival, age standardized estimates were derived using the ICSS weights; including patients with microscopically verified tumors and available information on age and stage and the respective local treatment; patients presenting with distant metastases or DCO notified tumors were excluded.

Table 5 shows 5-year RS and RER of BRC patients without distant metastases by age, tumor characteristics, and major systemic treatment options. Patients with nodal positive and HR positive or mixed tumors who did not receive chemotherapy and antiestrogen treatment had an age-standardized 5-year RS of 86% (RER 1.45; 95% CI: 0.78-2.68) compared to 99% for those who did. This survival difference was much more pronounced among patients aged ≥70 years than younger patients. Patients with positive lymph nodes but HR negative tumors who did not receive chemotherapy had a 5-year RS of 29% (2.89; 1.46-5.71) compared to 54% for patients obtaining chemotherapy.

**Table 5:** **Five year relative survival and relative excess risk of death of female breast cancer patients by tumor characteristics**, **systemic treatment and age**

Patient/tumor characteristics	Treatment	Provision	N	RS ^a^	SE	RER ^b^	95% CI	p-value
Nodal positive tumor, ER + or PgR+	Chemotherapy + antiestrogen treatment	yes	781	99.0	3.7	REF		
		no	506	86.2	3.1	1.45	0.78-2.68	0.239
Nodal positive tumor, ER + or PgR+, age 15–69 years	Chemotherapy + antiestrogen treatment	yes	701	92.0	1.8			
		no	264	88.3	3.0			
Nodal positive tumor, ER + or PgR+, age > =70 years	Chemotherapy + antiestrogen treatment	yes	80	95.0	8.9			
		no	242	83.5	5.5			
Nodal positive tumor, ER- and PgR-	Chemotherapy	yes	238	54.3	6.3	REF		
		no	30	28.5	9.7	2.89	1.46-5.71	0.002
Nodal negative tumor, ER- and PgR-	Chemotherapy	yes	310	94.9	5.5	REF		
		no	104	83.9	6.2	0.80	0.20-3.18	0.751
ER + or PgR+	Antiestrogen treatment	yes	3663	97.1	1.3	REF		
		no	536	85.6	3.8	1.75	0.99-3.07	0.053
ER + or PgR+, age 15–69 years	Antiestrogen treatment	yes	2664	96.8	0.7			
		no	401	89.4	2.4			
ER + or PgR+, age > =70 years	Antiestrogen treatment	yes	999	96.2	2.5			
		no	135	81.8	7.4			
HER2/neu positive tumor	Targeted therapy	yes	198	89.3	9.5	REF		
		no	509	84.4	4.1	2.17	0.91-5.14	0.080

Five year relative survival and relative excess risk of death of female breast cancer patients (ICD-10: C50) from Saarland estimated for calendar period 2005–2009 by tumor characteristics, systemic treatment and age. *N*: number of patients contributing survival experience; RS: point estimate of 5-year relative survival; *SE*: standard error of RS; *RER*: relative excess risk (of death); *CI*: confidence interval; a) except for age group-specific survival, age standardized estimates were derived using the ICSS weights; b) adjusted for age (15–49, 50–69, > = 70 years), tumor size (<5 cm (T1/T2), > = 5 cm (T3/T4)), lymph node involvement (negative, N1, N2/N3), histologic grade (G1/G2, G3/G4), hormone receptor status (ER + or PgR+, ER- and PgR-) and HER2/neu expression (positive, negative); including patients with microscopically verified tumors and available information on age, T, N, histologic grade, hormone receptor status, HER2/neu expression and provision of the respective systemic treatment; patients presenting with distant metastases or DCO notified tumors were excluded.

Survival of patients with nodal negative and HR negative tumors who did not receive chemotherapy was 84% (0.80, 0.20-3.18) compared to 95% if they underwent chemotherapy. Patients with HR positive or mixed tumors without antiestrogen treatment had a 5-year RS of 86% (1.75; 0.99-3.07) compared to 97% if such treatment was administered. The analysis of age specific 5-year RS of these patients showed a much higher survival difference in patients aged > =70 years, if they received no antiestrogen treatment (minus 14% units), than in younger patients (minus 8% units). Patients with HER2/neu expressed tumors who received no targeted therapy had a 5-year RS of 84% (2.17; 0.91-5.14) compared to 89% if trastuzumab was provided.

## Discussion

As a novelty, this study presents provision of cancer treatment and up-to-date survival of patients with non-metastasized BRC on a population level according to age, clinical subgroups, and major treatment options. The study revealed increasing adherence to CPG between 2000 and 2009, e.g. rises in the usage of BCS, chemotherapy and antiestrogen treatment and the introduction of SND for staging and use of trastuzumab in patients with HER2/neu expressed tumors. The analyses further demonstrated major disparities in the provision of cancer treatment by age.

The extent of adherence to CPG and the observed trends during the study period corresponded to findings from previous population-based studies from other Western countries (e.g. proportion of patients with BCS receiving radiotherapy: Saarland 2004/05: 85%, region of Piedmont (Italy) 2004: 88%, Canadian regions 2000–2004: ranging between 77% and 83%; chemotherapy in nodal positive patients: Saarland: 73%, region of Piedmont: 64%, Canadian regions: between 53%-68%; antiestrogen treatment in HR positive or mixed patients: Saarland 2000/2001: 79%, Canadian regions: 79%-85%) [38–41].

Compared to data from other studies including patients diagnosed prior to the millennium change, the observed proportions of guideline adherence were comparable or higher [42–47]. Previous studies from Germany on the extent of guideline adherence were based on a cohort study [48] or included hospital cohorts [42, 43, 49] and provided somewhat higher levels of guideline adherence and, along with a more favorable distribution of patient and tumor characteristics nominally higher survival estimates.

The implementation of specialized breast centers in Germany that aim at the implementation of evidence- and consensus-based CPG [50], which started in Saarland as well as in Germany in 2004 [51], are well reflected in the data. This particularly applies for the increased usage of systemic treatments (e.g. usage of chemotherapy in patients with nodal positive or HR negative tumors or trastuzumab in patients with HER2/neu expressed tumors increased by 19% units and 44% units to 79% and 47%, respectively, between 2000/01 and 2008/09). Dramatic changes in axillary staging could be observed after adoption of SND (usage 2000/01: 1%. 2008/09: 62%), a development that started somewhat earlier in the US [52].

Like previous studies from other countries, this study further demonstrated discrepancies in the adherence to CPG in elderly patients [53–56]. Other factors influencing provision of treatments include restricted access to cancer care, doctors’ perceptions, level of evidence, and patient preferences [57, 58], but also race, health insurance coverage, socio economic status and size of hospital may influence the receipt of guideline concordant cancer treatment [59].

Compared to other European or industrialized countries, Saarland ranks middle in terms of 5-year RS survival and its trends in the past [14, 60–62]. Inferior population-based survival of elderly patients has been reported in many previous studies (e.g. [12, 14, 63, 64]). Two recent studies showed similar age- and stage-stratified survival trends between Germany and the US among younger patients, but pronounced differences for elderly patients [65], or revealed inferior survival of elderly BRC patients across all clinical subgroups [15]. Inferior survival of elderly patients is commonly explained by comorbidity and differences in the delivery of cancer care [56, 66–69].

The analyses revealed inferior age standardized 5-year RS and tentatively increased RER of death for patients with nodal positive tumors, HR positive or mixed tumors and HER2/neu expressed tumors, if the patients did not receive recommended adjuvant chemotherapy with or without antiestrogen treatment (RER 1.45 and 2.89; p-values: 0.239 and 0.002), antiestrogen treatment (1.75; 0.053) and trastuzumab (2.17, 0.080). These results are in line with findings from few previous studies using cancer registry data [70] or data from hospital based cohorts [71, 72], although two recent registry-based studies did not find an association between guideline compliance and BRC survival [53, 54].

Commonly, population-based cancer registries record only a limited amount of data (such as age, tumor site, summary stage and morphology) which are used as surrogates for clinical information on tumor detection and treatment of the patients [73]. Thus survival estimates according to such surrogate information are representative for patients receiving “average” cancer care. The data available for this work allowed detailed analyses according to the actual provision of cancer treatment and survival of the BRC patients. The derived survival data then provide a quantitative measure of the effectiveness of guideline adherent cancer care in the routine setting and extend the data available from clinical trials. Often, clinical trials do not provide fully representative data on the effectiveness of cancer treatments due to selective inclusion of trial enrollees (e.g. under-representation of elderly patients) [74–76].

The study has several strengths. The completeness of case ascertainment of the Saarland Cancer Registry is regularly estimated above 95% [2, 18] and the registry regularly contributes to national and international collaborations [2, 62, 77]. Follow-up information was available for virtually all patients. As almost all tumors were microscopically, and as the used data were derived from multiple sources (e.g. discharge letters, case summaries, review of medical records at source by registry staff), the validity of tumor information and clinical data may be considered as high. By using period analysis methodology, most up-to-date estimates of cancer survival could be derived. Period estimates closely predict cancer survival later observed for patients diagnosed in the respective calendar period and allows early detection of changes in the long-term survival of patients, which has been shown by extensive empirical evaluation [28, 78–80].

However, a number of important limitations must be considered as well: first, even if the registry provided detailed data that exceed the amount usually available on a population level, the treatment information solely included whether a specific treatment was provided or not. Additional data on the start and end of a treatment, the intent or a premature discontinuation were not available. Neither was information available on comorbidity [81] and other factors, such as volume of health care providers [82, 83], or socio-economic status, [84–86] which are associated with the delivery of cancer care or disparities in cancer survival. It is therefore not possible to quantify to what extent the apparent increase in mortality associated with non-adherence to CPG is due to specific reasons for non-adherence, such as contraindications resulting from comorbidity, patient preferences or other reasons.

We used 5-year RS as a measure that corrects for mortality from other causes of death (and thus is a measure for net survival in the hypothetical situation, where BRC was the only cause of death). However, when comparing survival of BRC patients with regard to administered cancer treatment, comorbidity may act as a confounder. To address this issue (at least partly), adjustment for age (by means of stratification of the analyses and by deriving age-standardized estimates of 5-year RS) was used as information on comorbidity was not included in the used data.

Second, information on tumor characteristics and cancer treatment were available for some four out of five patients (e.g. HR status: 86%, local surgery: 77%, chemotherapy: 80%, antiestrogen treatment: 78%). Solely information on targeted therapy was available for 59% of the patients only. The extent of missing information and the observational design of the study warrant caution in the interpretation of the findings (which particularly applies to the association between provision of cancer treatment and outcome). For that reason, stratification of the analyses and age adjustment were applied to account (at least partly) for possible bias from exclusion of patients due to missing data.

Third, for some variables (e.g. age or co-variables in the modeled analyses) rather crude categories were used, but this allowed best use of the available data. Due to the distribution of clinical subtypes of BRC and the large proportion of patients already treated according to CPG, some strata were rather small and some of the derived estimates of 5-year RS and RER had large standard errors (>5% units) or p-values or age-specific analyses were even not possible for some subgroups of patients (e.g. patients with T3/T4 or HER2/neu positive tumors). Nevertheless, the derived estimates consistently revealed lower survival and tentatively increased RER of death of patients without guideline compatible local and adjuvant systemic treatment. Here, an analysis based on a much larger population would have allowed to extend the resolution of the analyses and to derive estimates with increased precision and would have increased the power of the analyses.

Within the National Cancer Plan, the nationwide implementation of hospital based cancer registries and the development of necessary information structures linking clinical information and data from population-based cancer registries has been launched in Germany in 2013 [87].

Currently, there is a time lag of about two and a half years after a calendar year has ended until case ascertainment and follow-up are sufficiently completed and the data are available for reporting and further research (this similarly applies to the Saarland Cancer Registry as well as other population-based cancer registries). The definition of specific episodes (e.g. tumor diagnosis, end of a specific treatment, or recurrence of the disease) that trigger a notification to the registry and the implementation of a common dataset for hospital based cancer registries and information structures for linkage of clinical data with population-based cancer registries will help to reduce the aforementioned latency.

A common database including both data from population-based cancer registries and data from hospital based cancer registries (to be built up in Germany in the years to come) will allow to use these data not only for survival studies that largely extend the possibilities presented in this study (e.g. providing cancer survival data adjusted for comorbidity, or analyses with regard to other endpoints or late effects of cancer treatment), but particularly for measuring the provision of cancer care at a much higher level of detail as a prerequisite to better understand the observed gaps in the provision of cancer care and to take action to overcome the observed survival deficits and increased cancer related mortality among patients with insufficient cancer care.

Even if Saarland constitutes only a small proportion of the national German population, it is well representative for Germany and its health care system and based on available high quality data, the findings of the study may nevertheless provide relevant evidence for clinicians and their patients, researchers and health care planners on BRC survival and cancer related mortality for Germany at this stage.

## Conclusions

Weighting up strengths and limitations, this study may provide important and clinically relevant findings. It reveals increasing adherence to major recommended treatment options, with high levels already observed for local treatment and substantial progress within recent years with regard to SND and adjuvant systemic treatment in a highly developed European country. It further provides population-based cancer survival for clinical subgroups of patients with regard to treatment usage and – based on the data available – demonstrated tentatively increased cancer related excess mortality among BRC patients who did not receive guideline adherent treatment. This study may thus provide relevant evidence for clinicians and their patients, researchers and health care planners of the effect of non-adherence to CPG on cancer related survival on a population level.

## Abbreviations

ALND: Axillary lymph node dissection
BCS: Breast conserving surgery
BRC: Breast cancer
CI: Confidence interval
CPG: Clinical practice guidelines
DCO: Death certificate only
HR: Hormone receptor
ICSS: International cancer survival standards
RER: Relative excess risk (of death)
RS: Relative survival
SND: Sentinel node dissection.
